# Supplementary material for: Spindle Position Checkpoint Kinase Kin4 Regulates Organelle Transport in Saccharomyces cerevisiae
Source: Biomolecules. 2023 Jul 10;13(7):1098. doi: 10.3390/biom13071098 (PMC10377308; doi:10.3390/biom13071098)
Supplement: Supplementary file 1 [file biomolecules-13-01098-s001.zip › biomolecules-2462708-supplementary.pdf]

This file includes legends for videos, supplementary Figures S1–S5, and Tables S2-S3.
It also includes raw western blots used in the manuscript.

Video 1. The *vps1Δdnm1Δ* cells mostly have single elongated peroxisomes. Time-lapse imaging analysis for the *vps1Δdnm1Δ* strain expressing genomically integrated mNG-PTS1 and cytosolic mCherry. Scale bar, 5 µm.

Video 2. The *vps1Δdnm1Δ* cells show a weak peroxisome segregation defect. Time-lapse imaging analysis for the *vps1Δdnm1Δ* strain expressing genomically integrated mNG-PTS1 and cytosolic mCherry. Scale bar, 5 µm.

Video 3. *KIN4* lacking *vps1Δdnm1Δ* cells show a strong peroxisome segregation defect. Time-lapse imaging analysis for the *vps1Δdnm1Δkin4Δ* strain expressing genomically integrated mNG-PTS1 and cytosolic mCherry. Scale bar, 5 µm.

Video 4. The *vps1Δdnm1Δinp2Δ* cells are defective in peroxisome inheritance. Time-lapse imaging analysis for the *vps1Δdnm1Δinp2Δ* strain expressing genomically integrated mNG-PTS1 and cytosolic mCherry. Scale bar, 5 µm.

Figure S1

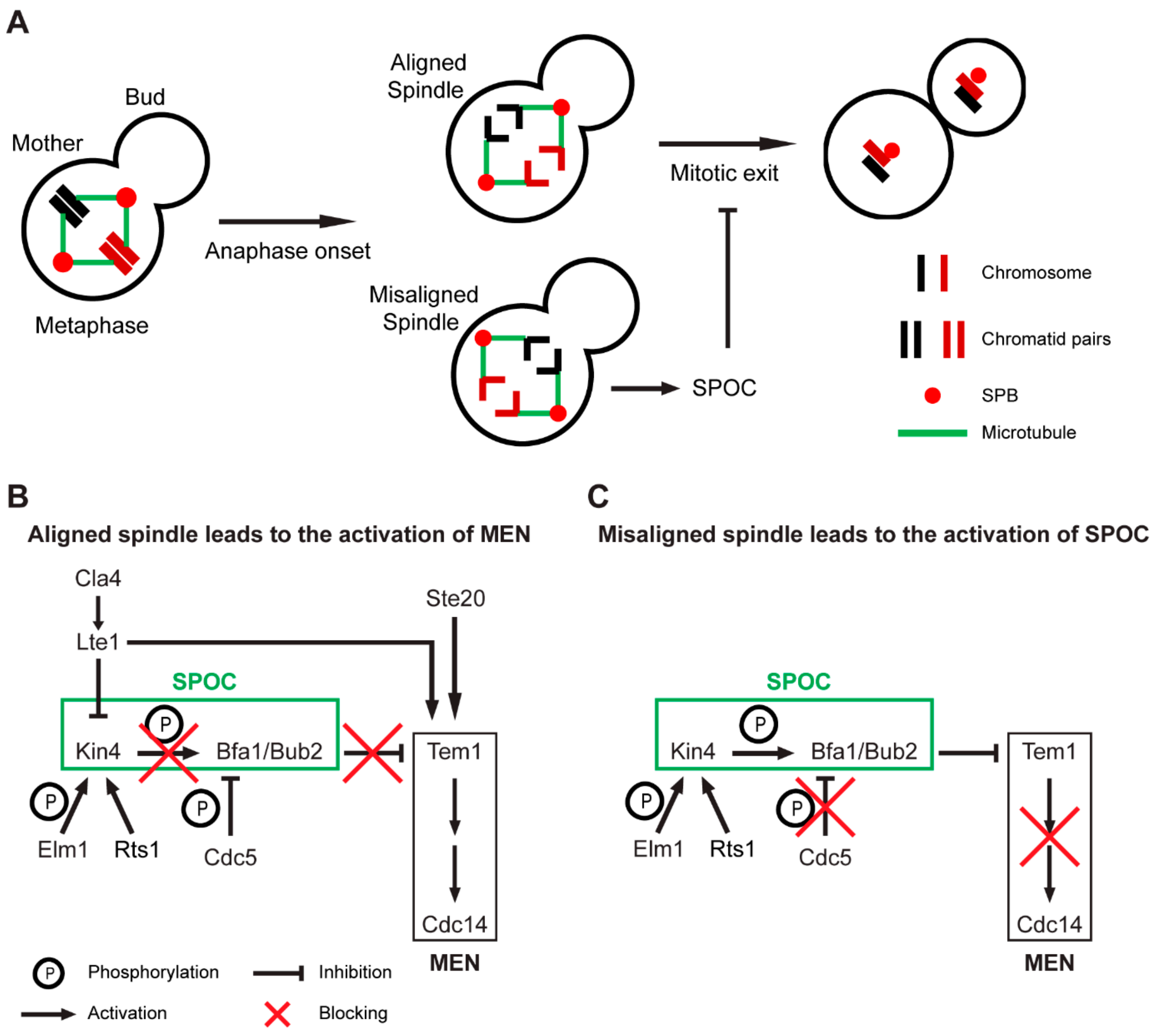

Figure S1. Schematic representation for the regulation of the SPOC and MEN pathways and the components involved. **(A)** The alignment of the mitotic spindle along the cell polarity axis around

the time of anaphase onset is crucial for faithful chromosome segregation and subsequent exit from mitosis. In the case of spindle misalignment, SPOC is activated to halt mitotic exit by negative regulation of the MEN. The two scenarios of aligned spindle and misaligned spindle and the spatio-temporal regulation of molecular players involved in the SPOC and MEN pathways are depicted in **(B)** and **(C),** respectively.

Figure S2

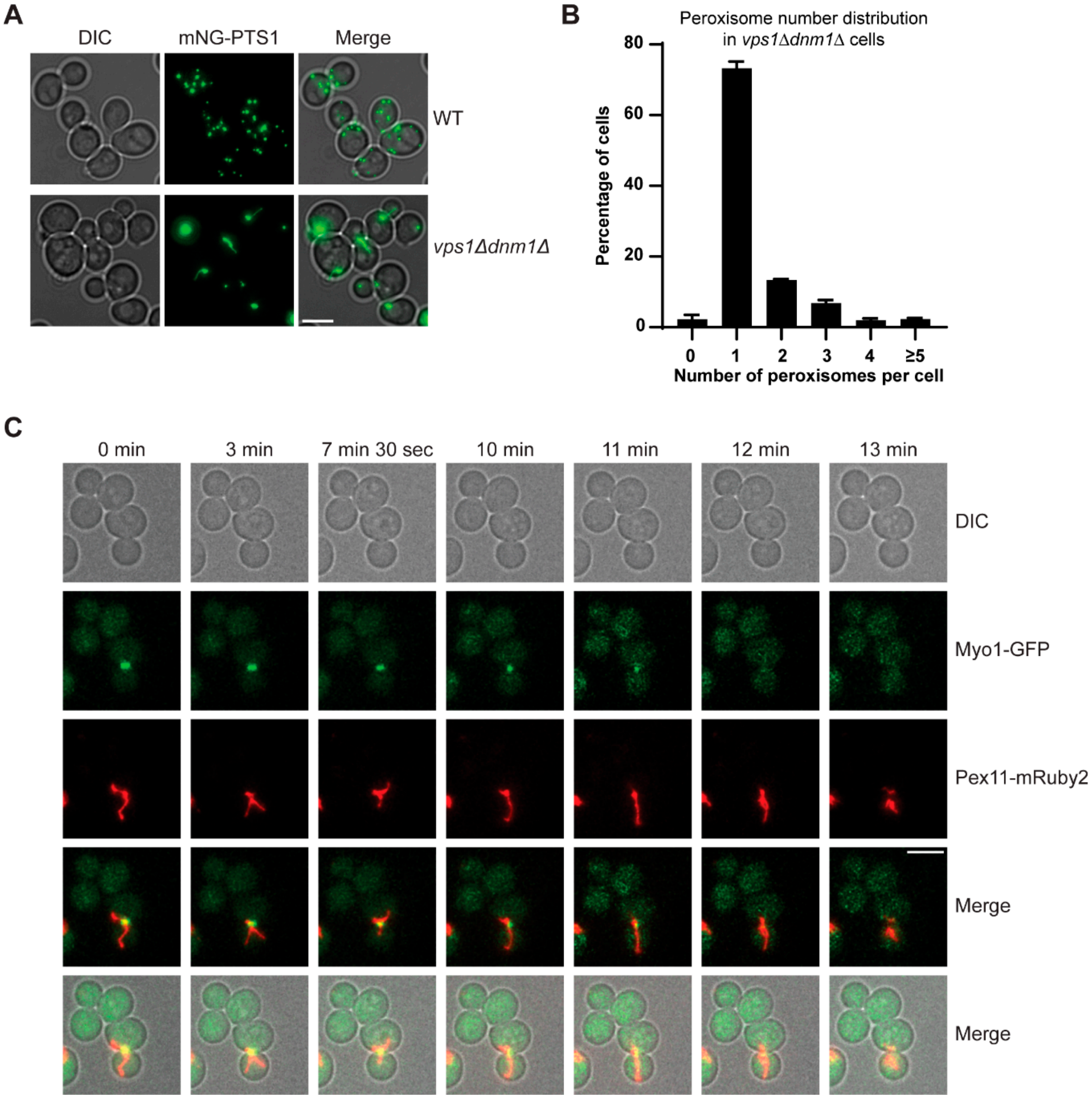

Figure S2. Peroxisome segregation in *vps1Δdnm1Δ* cells. **(A)** Epifluorescence images captured from the *S. cerevisiae* WT and *vps1Δdnm1Δ* cells expressing mNeonGreen fluorescent protein appended with a peroxisomal targeting signal type 1 (mNG-PTS1). Cells from exponentially growing cultures were used for epifluorescence microscopy experiments. Representative images are shown as merged Z-stacks. Bright-field images were collected in one plane and processed to highlight the cell circumference in blue. **(B)** Graph showing the distribution of peroxisome abundance for the *vps1Δdnm1Δ* strain indicated in (A). Quantitation was performed on a minimum of 100 cells from 3 independent experiments and the error bars indicate standard deviation. **(C)** Time-lapse images of *vps1Δ/dnm1Δ* cells around the time of the cytokinesis, where Pex11-mRuby2 and Myo1-GFP are a peroxisomal marker and an AMR marker respectively. Scale bar, 5 μm.

Figure S3

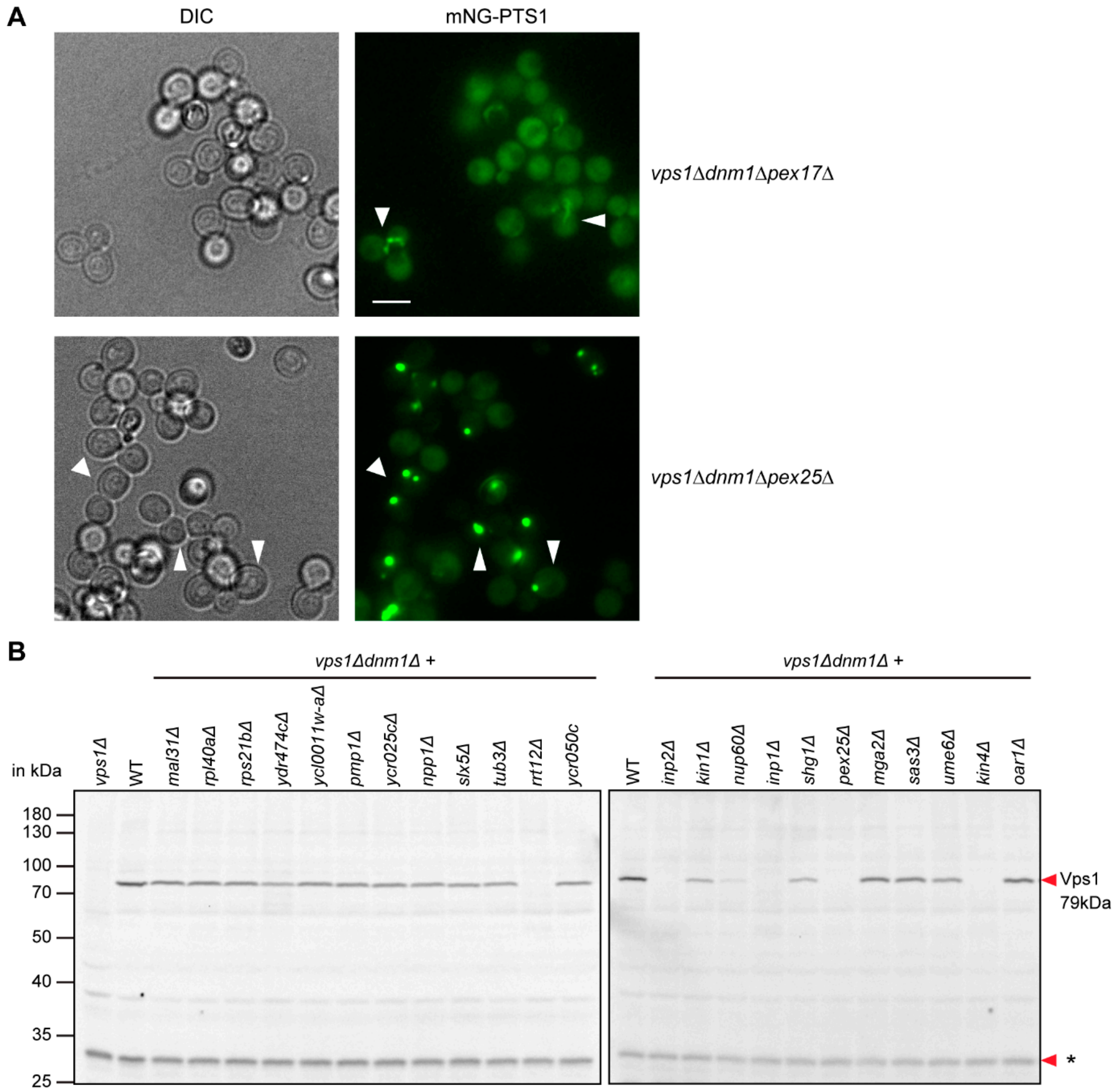

Figure S3. Analysis and validation for screen mutants. **(A)** Representative images for *vps1Δdnm1Δpex17Δ* and *vps1Δdnm1Δpex25Δ* cells from the genome-wide screen. The white arrowheads indicate cells showing partial mNG-PTS1 localisation to peroxisomes in *vps1Δdnm1Δpex17Δ* and cells with segregation defect in *vps1Δdnm1Δpex25Δ*. Cells from exponentially growing cultures were used for epifluorescence microscopy experiments. Scale bar, 5 µm. **(B)** Western blot analysis for lysates from Class 3 category mutants to identify false positives. The cell extracts were isolated by TCA precipitation and tested with the anti-Vps1 antibody to check the presence of Vps1 protein. * Indicates the background band and is a loading control.

Figure S4

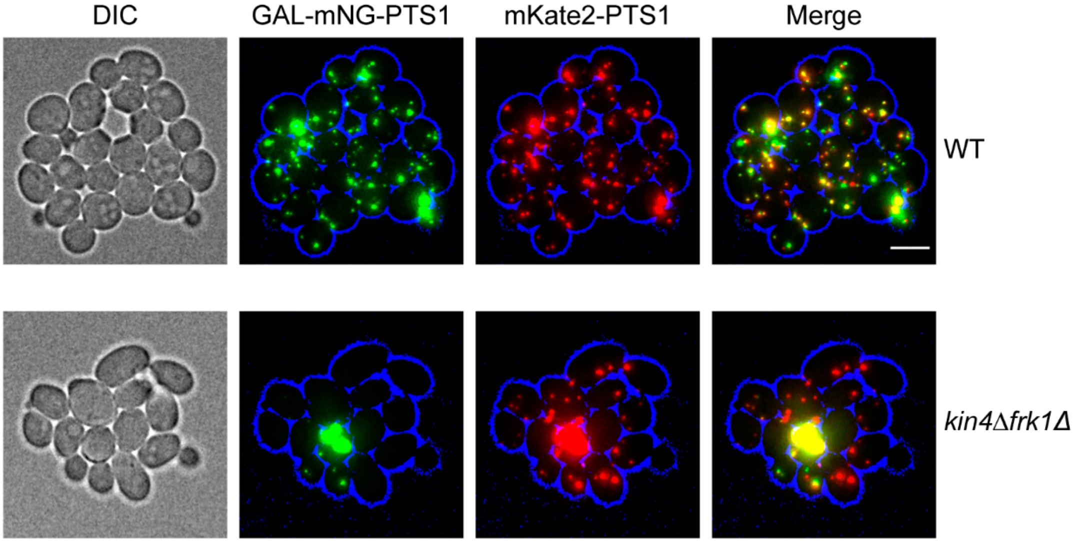

Figure S4. *kin4Δfrk1Δ* cells lacking peroxisomes form them *de novo*. Wild type and *kin4Δfrk1Δ* cells constitutively expressing mKate2-PTS1 and conditionally expressing mNG-PTS1 were grown for 2.5 h on galactose medium and chased for 2 h on glucose medium. Cells were then seeded thinly under a glucose-containing agarose pad in an imaging μ-dish (Ibidi) and allowed to grow for 6-8 h before imaging so that single budding cells can give rise to a colony. If any peroxisomes are formed *de novo* after the shutdown of mNG-PTS1 expression, these peroxisomes will be labelled with mKate2-PTS1 only. In wild-type cells, all peroxisomes are labelled with red and green indicating that peroxisomes are actively dividing and segregating. In contrast, in the *kin4Δfrk1Δ* colony, there are some cells with red peroxisomes that lack any mNG signal. These peroxisomes have been formed *de novo*.

Figure S5

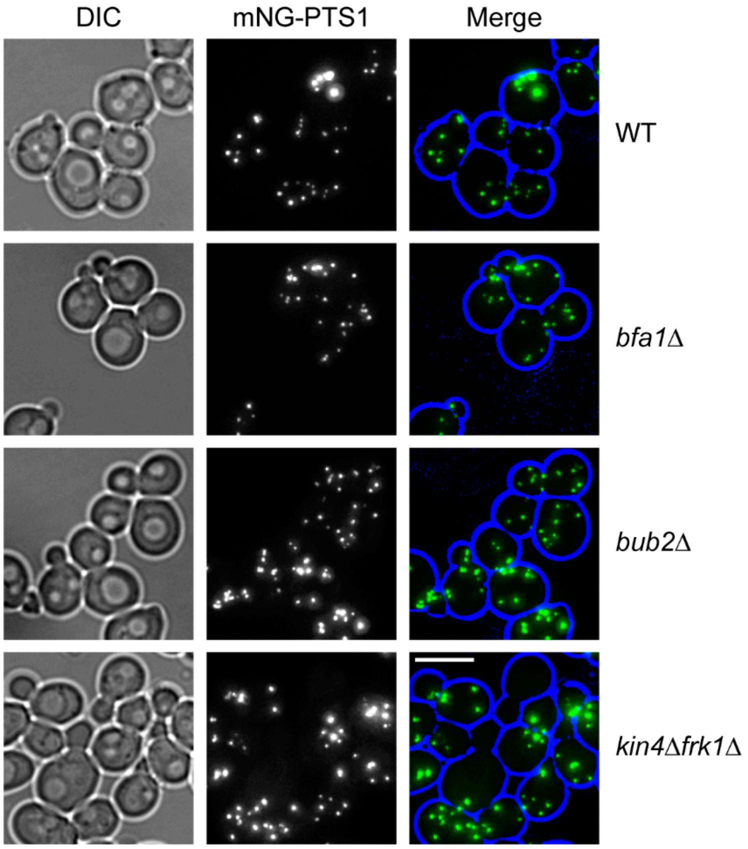

Figure S5. The *bfa1Δ* and *bub2Δ* cells do not show peroxisome inheritance defects. Epifluorescence images were captured from exponentially growing *S. cerevisiae* WT, *bfa1Δ*, *bub2Δ*, and *kin4Δfrk1Δ* cells expressing mNG-PTS1. Representative images are shown as merged Z-stacks. Bright-field images were collected in one plane and processed to highlight the cell circumference in blue. Scale bar, 5 μm.

Table S2. A list of yeast strains used in this study.

Strain and genotype	Reference
*BY4741 MAT**a** his3Δ1 leu2Δ0 met15Δ0 ura3Δ0*	EUROSCARF
BY4742 MATα his3Δ1 leu2Δ0 lys2Δ0 ura3Δ0	EUROSCARF
BY4742 dnm1Δ::kanMX4 vps1Δ::loxP	[43]
BY4742 dnm1Δ::kanMX4 vps1Δ::loxP MYO1::MYO1-GFP-hphMX4 PEX11:PEX11-mRuby2-his3MX6	This study
SGA strain (YEH738) MATα leu2Δ0 lys2Δ0 ura3Δ0 his3Δ1::TEF2pr-mCherry-URA3 can1Δ::STE2pr-spHIS5 lyp1Δ::STE3pr-LEU2 dnm1Δ::MET15 vps1Δ::mNG-PTS1-natMX6	This study
YEH738 pex17Δ::kanMX4	This study
YEH738 pex25Δ::kanMX4	This study
YEH738 inp1Δ::kanMX4	This study
YEH738 inp2Δ::kanMX4	This study
YEH738 kin4Δ::kanMX4	This study
YEH738 kin4Δ::kanMX4 inp1Δ::hphMX4	This study
YEH738 kin4Δ::kanMX4 inp2Δ::hphMX4	This study
YEH738 bfa1Δ::kanMX4	This study
YEH738 bub2Δ::kanMX4	This study
YEH738 rts1Δ::kanMX4	This study
BY4742 dnm1Δ::kanMX4 vps1Δ::loxp kin4::hphMX4	This study
BY4742 dnm1Δ::kanMX4 vps1Δ::loxP frk1Δ::natMX6	This study
BY4742 dnm1Δ::kanMX4 vps1Δ::loxP kin4::hphMX4 frk1Δ::natMX6	This study
BY4742 inp1Δ::kanMX4	EUROSCARF
BY4742 inp2Δ::kanMX4	EUROSCARF
BY4741 kin4Δ::kanMX4	EUROSCARF
BY4741 frk1Δ::kanMX4	EUROSCARF
BY4742 frk1Δ::kanMX4 kin4::hphMX4	This study
BY4741 MYO2::MYO2-3HA-his3MX6	This study
BY4741 frk1Δ::kanMX4 kin4Δ::hphMX4 MYO2::MYO2-3HA-his3MX6	This study
BY4741 bfa1Δ::kanMX4	EUROSCARF
BY4741 elm1Δ::kanMX4	EUROSCARF
BY4741 bub2Δ::kanMX4	EUROSCARF
BY4742 frk1Δ::kanMX4 kin4::hphMX4 inp2Δ::natMX6	This study

Table S3. A list of plasmids used in this study.

Plasmid Name	Vector backbone	Promoter	Insert	UniProt ID	Source
pAUL4	Ycplac111	*HIS3*	*mNG-PTS1*		Lab stock
pAS5	Ycplac33	*HIS3*	*HcRed-PTS1*		Lab stock
pAUL28	Ycplac33	*HIS3*	*mKate2-PTS1*		Lab stock
pEW318	Ycplac33	-	-		Lab stock

pEW319	Ycplac111	-	-		Lab stock
pLE50	Ycplac33	*GAL1*	*KIN4*	Q01919	This study
pLE52	Ycplac111	*GAL1*	*KIN4*	Q01919	This study
pLE58	Ycplac111	*KIN4*	*KIN4-GFP*	Q01919	This study
pLE60	Ycplac111	*KIN4*	*KIN4-T209A-GFP*	Q01919	This study
pLE51	Ycplac33	*GAL1*	*FRK1*	Q03002	This study
pLE53	Ycplac111	*GAL1*	*FRK1*	Q03002	This study
pLE107	Ycplac33	*INP2*	*INP2-2xProtA*	Q03824	This study
pLE49	Ycplac111	*INP2*	*INP2-GFP*	Q03824	This study
pLE120	Ycplac33	*INP2*	*INP2-mNG*	Q03824	This study
pAA3	Ycplac33	*INP2*	*INP2ΔMIS-mNG*	Q03824	This study
pAA4	Ycplac33	*INP2*	*INP2ΔMIS-2xProtA*	Q03824	This study
